# *S100A9* plays a key role in *Clostridium perfringens* beta2 toxin-induced inflammatory damage in porcine IPEC-J2 intestinal epithelial cells

**DOI:** 10.1186/s12864-023-09118-6

**Published:** 2023-01-12

**Authors:** Jie Li, Kaihui Xie, Jiaojiao Yang, Juanli Zhang, Qiaoli Yang, Pengfei Wang, Shuangbao Gun, Xiaoyu Huang

**Affiliations:** 1 College of Animal Science and Technology, Gansu Agricultural University, Lanzhou, 730070 China; 2 College of Life Sciences, Longdong University, Qingyang, 745000 China; 3 Gansu Research Center for Swine Production Engineering and Technology, Lanzhou, 730070 China

**Keywords:** Piglet diarrhea, *Clostridium perfringens* type C, RNA-Seq, *S100A9* target gene, Functional verification

## Abstract

**Background:**

As an important regulator of autoimmune responses and inflammation, *S100A9* may serve as a therapeutic target in inflammatory diseases. However, the role of *S100A9* in *Clostridium perfringens* type C infectious diarrhea is poorly studied. The aim of our study was to screen downstream target genes regulated by *S100A9* in *Clostridium perfringens* beta2 (CPB2) toxin-induced IPEC-J2 cell injury. We constructed IPEC-J2 cells with *S100A9* knockdown and a CPB2-induced cell injury model, screened downstream genes regulated by *S100A9* using RNA-Seq technique, and performed functional enrichment analysis. The function of *S100A9* was verified using molecular biology techniques.

**Results:**

We identified 316 differentially expressed genes (DEGs), of which 221 were upregulated and 95 were downregulated. Functional enrichment analysis revealed that the DEGs were significantly enriched in cilium movement, negative regulation of cell differentiation, immune response, protein digestion and absorption, and complement and coagulation cascades. The key genes of immune response were *TNF*, *CCL1*, *CCR7*, *CSF2*, and *CXCL9*. When CPB2 toxin-induced IPEC-J2 cells overexpressed *S100A9*, Bax expression increased, Bcl-2 expression and mitochondrial membrane potential decreased, and SOD activity was inhibited.

**Conclusion:**

In conclusion, *S100A9* was involved in CPB2-induced inflammatory response in IPEC-J2 cells by regulating the expression of downstream target genes, namely, *TNF*, *CCL1*, *CCR7*, *CSF2*, and *CXCL9*; promoting apoptosis; and aggravating oxidative cell damage. This study laid the foundation for further study on the regulatory mechanism underlying piglet diarrhea.

**Supplementary Information:**

The online version contains supplementary material available at 10.1186/s12864-023-09118-6.

## Introduction

Piglet diarrhea is a disease caused by various pathogens. It is the main cause of piglet mortality in pig farming [1], which leads to massive economic losses for the global pig industry. Among the most common pathogens causing piglet diarrhea is *Clostridium perfringens* type C. In most mammals, infection with *Clostridium perfringens* causes inflammation, and the toxins produced in the intestine can enter the systemic circulatory system, causing toxemia and animal death [2]. *Clostridium perfringens* type C produces the highly virulent CPB2 toxin, which causes necrotizing enteritis in animals and is considered to be the main pathogenic factor [3–5]. Currently, the treatment and prevention of piglet diarrhea is a difficult task in pig farming. The importance of good housing conditions and hygiene is essential to prevent diseases in pigs. In addition, the main use of antibiotics in animal production is for the treatment and prevention of diseases, but also as growth promoters [6, 7]. Antibiotics are effective in treating piglet diarrhea [8]; however, antibiotic residues seriously affect food safety and pose a threat to humans after consuming such foods. Antibiotic misuse and resistance affects global health [9]. In 2006, the European Union completely bans the use of antibiotics in animal production [8]. After this legal regulations, it has become more difficult to treat piglet diarrhea. One Health approach believes that the health of humans, animals and the environment are closely interrelated and interdependent[10]. Antibiotic resistance (ABR) is considered a key One Health issue. Since many other countries outside the EU have different legislation on antimicrobial use, it makes sense to address ABR through a One Health approach [11]. Therefore, finding alternatives to antibiotics for the treatment and prevention of piglet diarrhea is essential for healthy and sustainable livestock industry.

Calcium-binding protein S100A9 is reported to be involved in inflammation [12]. Several studies have demonstrated that *S100A9* is a biomarker of inflammatory response and can induce an inflammatory cascade [13, 14]. *S100A9* is reported to activate neutrophils to participate in the immune response as a target of inflammation in autoimmune diseases [15]. In addition, *S100A9* acts as a ligand for late glycosylation end-products and regulates the development of inflammatory responses [16]. S100 protein family regulates physiological functions such as cell proliferation, differentiation, metabolism and signal transduction [17]. Upregulated *S100A9* predicts poor prognosis in patients with cancer [17]. *S100A9* expression is significantly upregulated in patients with inflammatory bowel disease [18]. In summary, the expression of *S100A9*, an early participant in the development of inflammatory process, correlates with the severity of inflammation and is critical for the development of inflammatory diseases caused by bacterial pathogens. However, studies on *S100A9* in *Clostridium perfringens* type C piglet diarrhea are scarce.

In our previous study, we reported that *S100A9* is an important factor for diarrhea caused by *Clostridium perfringens*, based on a high-throughput sequencing approach called Illumina Hiseq 4000 [19]. In this study, we constructed IPEC-J2 cells with *S100A9* knockdown and screened the downstream key target genes regulated by *S100A9* with transcriptome sequencing. Using molecular biology techniques, we studied the effect of *S100A9* on CPB2-induced IPEC-J2 cell apoptosis and inflammatory injury. This study provided a research basis for the further exploring on the mechanism of action of *S100A9* in regulating inflammatory response in porcine diarrhea.

## Materials and methods

### Tissue sample collection

Tissue samples were collected from 7-day-old piglets (Landrace × Yorkshire; *n* = 5) after slaughter. All samples were immediately put into liquid nitrogen for long-term storage in an ultra-low temperature refrigerator at − 80 °C.

### Preparation of CPB2 toxin

The CPB2 toxin was prepared according to the method described by Gao [20] and Luo [21]. The extracted CPB2 was eluted with various concentrations of imidazole. The protein bands were detected using SDS-PAGE. The single toxin protein in the band was treated with ToxOut™ Rapid Endotoxin Removal Kit (BioVision, USA). CPB2 was stored at − 80 ℃.

### Cell culture, transfection, and toxin handling

The porcine IPEC-J2 cell line (intestinal epithelial cells) was purchased from Beina Biotechnology (Beijing, China). Cells were grown in DMEM medium containing 10% fetal bovine serum, 100 U/mL penicillin, and 100 mg/L streptomycin at 37 ℃ with 5% CO_2_.

For molecular biology experiments, the cells were divided into blank control (Control) and CPB2 treated (CPB2) groups, *S100A9* overexpression (CPB2 + pc-*S100A9*) and negative control (CPB2 + pcDNA3.1) groups, and *S100A9* interference (CPB2 + si-*S100A9*) and negative control (CPB2 + si-NC) groups. The pc-*S100A9*, pcDNA3.1, si-*S100A9*, and si-NC vector were transfected into IPEC-J2 cells using Lipofectamine® 2000 Reagent (Invitrogen, CA, USA) as per the manufacturer’s instructions. After attaining 80% confluency after transfection, the cells were cocultured with 20 μg/mL CPB2 for 24 h. Finally, cells were collected for subsequent experiments. The pcDNA3.1 was purchased from Thermo Fisher Scientific, and the *S100A9* overexpression vector pc-*S100A9* was constructed by cloning the coding region of *S100A9* (NM_001177906.1) into the pcDNA3.1 vector using Nhel and XhoI enzyme sites. The pc-*S100A9*, si-NC and si-*S100A9* (5′-UUCUCCGAACGUGUCACGUTT-3′) were designed and synthesized by GenePharma (Shanghai, China).

### Library construction and RNA-Seq

The total RNA was extracted from cell samples with TRIzol reagent (Invitrogen, CA, USA). The RNA was purified with RNA clean kit (TIANGEN, PD412, Beijing, China) according to the manufacturer's instructions, and RNA content and purity of the cell samples were measured with NanoDrop ND-1000 (NanoDrop, Wilmington, DE, USA). We evaluated the integrity of RNA using Bioanalyzer 2100 (Agilent, CA, USA), where RIN > 7.0 is consistent with downstream experiments. The RNA integrity was also confirmed by electrophoresis with denaturing agarose gel. In two rounds, Dynabeads Oligo (dT)25–61,005 (Thermo Fisher, CA, USA) was used to purify poly(A) RNA from 1 μg total RNA. Using the Magnesium RNA Fragmentation Module (NEB, cat.e6150, USA), poly(A) RNA was fragmented and reverse-transcribed using the SuperScriptTMII Reverse Transcriptase (Invitrogen, cat.1896649, USA) to generate cDNA. Next, a paired-end cDNA library was created with an average insert size of 300 ± 50 bp. In accordance with the standard protocol, we used the Illumina Novaseq™ 6000 (LC Bio Technology CO., Ltd. Hangzhou, China) to perform double-end sequencing.

### Bioinformatic analysis

The raw data was downlinked in fastq format. To obtain clean data, the Cutadapt software (V1.9) was used to remove adaptor reads, reads containing N proportion > 5%, and low-quality reads. Clean reads were mapped to the genome (*Sus scrofa* Ensembl v96) using HISAT2 (V2.0.4) [22]. Genes or transcripts assembling was performed using StringTie (V1.3.4d) [23], followed by comparison of transcripts with reference annotations using gffcompare (V0.9.8) software to obtain final assembly annotation results. The R package DESeq2 was used to analyze significant variations across samples [23]. The genes with |log_2_(fold change)|≥ 1 and *p*-value < 0.05 were defined as differentially expressed genes (DEGs) and were enriched in Gene Ontology (GO) [24] and Kyoto Encyclopedia of Genes and Genomes (KEGG) [25].

### GO and KEGG enrichment analyses

GO has three ontologies, namely, molecular function (MF), cellular component (CC), and biological process (BP) to describe a gene [26]. GO terms of top 25, top 15, and top 10 up- and downregulated DEGs were selected to plot GO enrichment histograms. The up- and downregulated DEGs were used for GO and KEGG pathway enrichment analyses. A scatter plot of the results of top 20 with the smallest *p*-value (most significantly enriched) was created based on the enrichment analysis results.

### Protein–protein interaction (PPI) network analysis of DEGs

The interactions among the 316 DEGs screened above were studied using the String database [27]; *Sus scrofa* was used as the organism for the interaction analysis. The resulting data in TSV format were exported. Cytoscape (V3.8.0) was used to create a PPI network of DEGs. The MCODE plugin was used to analyze protein clusters with a higher degree of association across the PPI network. CytoNCA plugin was used for PPI network centrality analysis and evaluation, and the BC score of each node was calculated using Betweenness Centrality (BC) as the scoring criterion. The MCC algorithm was used to filter out the hub genes.

### Assessment of differential mRNA levels using RT-qPCR

Overall, 12 DEGs (6 upregulated and 6 downregulated genes) were randomly selected from the RNA-Seq data to verify their authenticity. As an internal control, *GAPDH* was used. Primer synthesis was performed by Zhongke Yutong Biotechnology (Shanxi, China). The primer information is given in Table 1. RNA was reverse-transcribed into cDNA using Evo M-MLV RT Kit (Accurate Biotechnology, Hunan, China). RT-qPCR was performed using 2 × Universal Blue SYBR Green qPCR Master Mix (Servicebio, Wuhan, China) on the LightCycler 480II instrument (Roche, Switzerland) as per the following conditions: 95 °C for 30 s, 95 °C for 5 s, and 60 °C for 30 s; 40 cycles. All experiments were performed using three replicates. We used the2^−ΔΔCt^ method [28] to calculate the mRNA levels.

**Table 1:** Primer sequences for RT-qPCR

Gene name	Primer sequence (5ʹ-3ʹ)	Product length (bp)	Accession no
*CCR7*	F: GATGGTGGTGGGCTTTCTGA	112	NM_001001532.3
	R: CACCTTGATGGCCTTGTTGC		
*PLK2*	F: CAGTCAAGTGACGGTGCTGA	116	XM_003133981.5
	R: ACTGAAGGAGGTAGAGCCGA		
*TNF*	F: GGCCCAAGGACTCAGATCAT	82	NM_214022.1
	R: CTGTCCCTCGGCTTTGACAT		
*CCN1*	F: GCTCAAAGACCTGCGGAACT	183	XM_021094769.1
	R: AAGTGAACTTGACCGGCTCG		
*CSF2*	F: CTCCCACTGACAGAGCCAAA	118	NM_214118.2
	R: AGGCCTGTATCAGGGTCAAC		
*NEK5*	F: AAGGTCATTGGGGAAGGTGC	209	XM_005668386.3
	R: AACAGCCTGCCGTTCTCTTG		
*CXCL9*	F: GACTCAGTGGAACACCTACAGA	142	NM_001114289.2
	R: TGCAGGAACAACGTCCATTC		
*ANGPT1*	F: GGATATACTGAGAGGAGGGTGC	128	XM_021088688.1
	R: GACAGTTCCCGTCGTGTTCT		
*CCL1*	F: GAGCATGCATGTGTCGTCCT	213	NM_001166491.1
	R: CAACTCGGGCAGGGCTTTAT		
*GC*	F: TGAAAGCGACTCCCCATTCC	140	XM_003356971.4
	R: CGTCATTTGTTGGCTCCACG		
*CCL5*	F: ACACCACACCCTGCTGTTTT	168	NM_001129946.1
	R: TGTACTCCCGCACCCATTTC		
*IL11*	F: GCTGAACCTGACGCTTGACT	101	XM_021095008.1
	R: GAACTGGCTTTGAAGGACGC		
*S100A9*	F: GGGACACCCTGAACCAGAAA	193	NM_001177906.1
	R: TCCTCGTGAGAAGCTACCGT		
*GAPDH*	F: AGTATGATTCCACCCACGGC	139	NM_001206359.1
	R: TACGTAGCACCAGCATCACC		

### Western blot analysis

IPEC-J2 cells were lysed on ice with RIPA lysis solution. BCA Protein Assay Kit (Bioss, China) was used to detect and determine the total protein concentration. The protein samples were loaded with 5 × SDS-PAGE loading buffer and separated using 10% SDS-PAGE after denaturation of the protein. The protein bands were transferred to PVDF membrane. The PVDF membrane were blocked with 5% skimmed milk for 1 h and further incubated with 1:1000 dilution of primary antibodies overnight at 4 °C. The primary antibodies were S100A9 (PROGEN, Heidelberg, Germany), Bax (Bioss, China), Bcl-2 (Bioss, China), and β-actin (Bioss, China). Next, the membranes were incubated with secondary antibodies [HRP-labeled goat anti-rabbit (1:3000, Servicebio, Wuhan, China) or HRP-labeled goat anti-mouse (1:3000, Servicebio, Wuhan, China)] for 2 h at 37 °C on a shaker and washed 5 times with TBST. The bands were visualized using ECL luminescent solution (Absin, Shanghai, China) and imaged using Fusion FX system (VILBER, France).

### Detection of changes in mitochondrial membrane potential in IPEC-J2 cells using JC-1 probe assay

In a 24-well plate, cells in logarithmic growth phase were seeded at a density of 1 × 10^6^ cells/mL, transfected with Lipofectamine® 2000 reagent (Invitrogen, CA, USA) for 24 h, and further treated with CPB2 toxin for 24 h. Mitochondrial membrane potential was assessed using Mitochondrial membrane potential assay kit with JC-1 (Beyotime, Shanghai, China) according to the manufacturer’s instructions. In brief, 200 μL of cell culture medium and 200 μL of JC-1 staining solution was added to each well of a 24-well plate, mixed thoroughly, and incubated at 37 °C for 20 min with 5% CO_2_. Finally, the JC-1 staining working solution was discarded and the cells were washed 3 times using JC-1 staining buffer; 500 μL of cell culture medium was added, and a fluorescent inverted microscope (Olympus, Japan) was used for observation and photography.

### Assessment of apoptosis using Hoechst 33,258 staining

IPEC-J2 cells were cultured on 24-well plates at a density of 1 × 10^6^ cells/mL and were treated with transfection with Lipofectamine® 2000 reagent (Invitrogen, CA, USA) and inoculation for 24 h, respectively. Further, 300 μL of Hoechst 33,258 staining solution (Solarbio, Beijing, China) was added to the cells. The cells were incubated for 30 min in dark. Further, the staining solution was discarded. The cells were washed 3 times with PBS or culture medium and observed and photographed under a fluorescent inverted microscope.

### Superoxide dismutase (SOD) activity assay

Cells cultured in 24-well plates were separately transfected and incubated with CPB2 for 24 h. Further, the cell culture medium was removed, and the cells were washed 3 times with PBS, followed by the addition of 200 µL SOD per well to fully lyse the cells. The suspension was centrifuged at 4 °C at 12,000 rpm for 3–5 min, and the supernatant was taken for the analysis. SOD activity was detected using the Total SOD Activity Assay Kit (Beyotime, Shanghai, China) as per the manufacturer’s instructions. After incubation for 30 min at 37 °C, the absorbance was measured at 450 nm.

### Statistical analysis

Data processing was performed using SPSS 19.0 software. Student’s t-test was used for the comparisons between two groups, and one-way ANOVA was used for the comparisons among multiple groups. All experiments were performed three times. Data were expressed as mean ± SD. The graphs were plotted using GraphPad Prism 8.0. *P* < 0.05 considers the difference to be significant, *P* < 0.01 considers the difference to be extremely significant.

## Results

### Assessment of *S100A9* expression

**Fig. 1:**
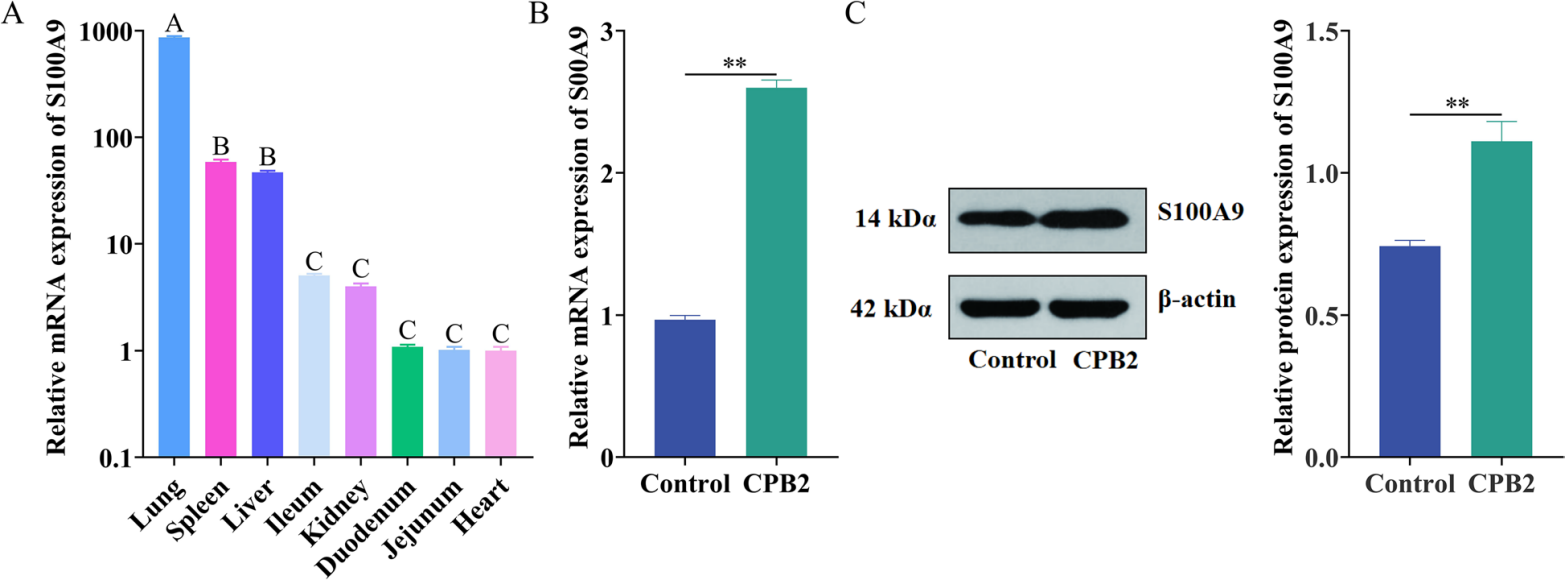
Assessment of mRNA and protein levels of *S100A9*. **A** Tissue profile of *S100A9 S100A9* mRNA levels. **B** *S100A9* mRNA levels in IPEC-J2 cells. **C** Protein levels of S100A9 in IPEC-J2 cells. Note: The same capital letters indicate insignificant differences (*P* > 0.05); different capital letters indicate significant differences (*P* < 0.05). *P < 0.05, **P < 0.01.

RT-qPCR revealed the presence of mRNA levels of *S100A9* in the lung, spleen, liver, ileum, kidney, duodenum, jejunum, and heart tissues of piglets (Fig. 1A). The highest mRNA level of *S100A9* was observed in the lung, followed by the liver and spleen. The lowest mRNA level of *S100A9* was in the heart. Further, IPEC-J2 cells were treated with 20 μg/mL CPB2 for 24 h, and total cellular RNA was extracted. RT-qPCR revealed that mRNA levels of *S100A9* were significantly upregulated (*P* < 0.01) (Fig. 1B). Western blot (Fig. 1C, Supplementary Fig. 1) revealed that the expression of *S100A9* was higher in the CPB2 group than in the control group (*P* < 0.01). It was speculated that *S100A9* plays an important role in the CPB2-induced inflammatory response in IPEC-J2 cells.

### Quality control of sequencing data

To further screen the key genes regulating the CPB2-induced inflammatory response in IPEC-J2 cells, three IPEC-J2 cell samples each from the CPB2 + si-NC and CPB2 + si-*S100A9* groups were collected to construct six cDNA libraries. The RNA-Seq results are given in Table 2. On an average, 52,793,825 and 54,079,491 raw reads were generated from cDNA libraries constructed from the CPB2 + si-NC and CPB2 + si-*S100A9* groups, respectively. The valid ratio (%) was 94.38% and 95.10%, respectively. Q20% were 99.93% for both groups, and Q30% were 97.59% and 97.54%, respectively, with GC content > 51%.

**Table 2:** Sequencing data quality control

Sample	Raw reads	Valid reads	Valid ratio (%)	Q20%	Q30%	GC content (%)
CPB2 + si_NC_1	55,604,212	52,016,636	93.55	99.92	97.51	51
CPB2 + si_NC_2	50,762,526	48,098,858	94.75	99.93	97.6	51.5
CPB2 + si_NC_3	52,014,736	49,328,396	94.84	99.94	97.67	51.5
CPB2 + si_S100A9_1	54,383,542	51,840,580	95.32	99.93	97.58	51.5
CPB2 + si_S100A9_2	55,132,804	52,306,692	94.87	99.92	97.54	51
CPB2 + si_S100A9_3	52,722,128	50,138,370	95.1	99.93	97.51	51

### Analysis of DEGs

**Fig. 2:**
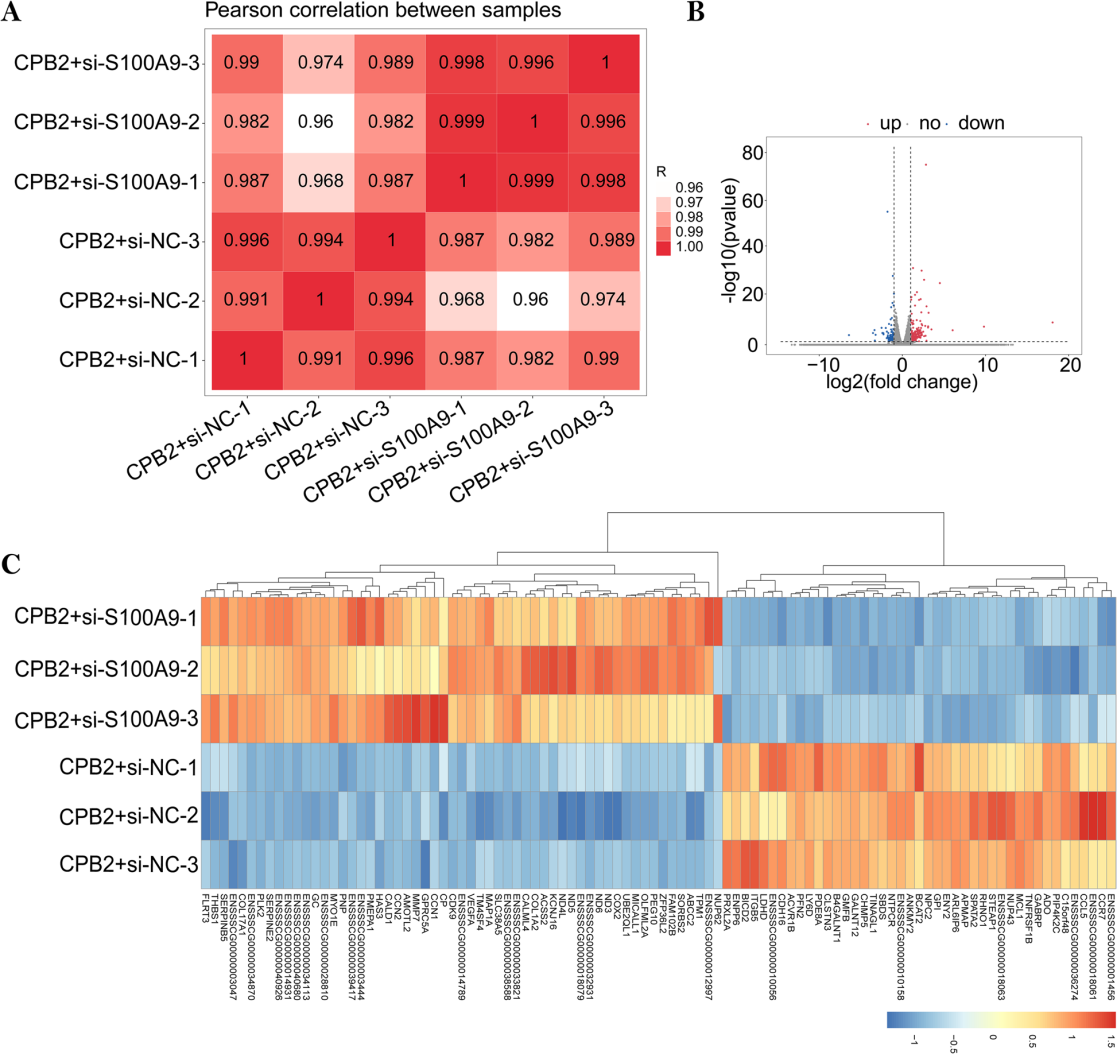
Analysis of DEGs. **A** The correlation analysis of expression in samples. The larger the correlation coefficient between samples, the better the sample clustering. **B** The volcano plot of differential gene expression levels. The red, blue, and grey dots represent upregulated, downregulated, and non-significant DEGs, respectively. **C** Cluster analysis of DEGs. The heat map was created by taking the top 100 genes with the smallest p-value as a starting point. Red and blue indicates genes that were relatively over- or underexpressed, respectively

The RNA-Seq results showed that the R^2^ values of both samples were > 0.96 (Fig. 2A), and the expression patterns of the two groups were highly similar. This indicated that the sequencing was reliable and can be used for subsequent bioinformatic analysis. Using |log_2_(fold change)|≥ 1 and p-value < 0.05 as the screening criteria, 316 DEGs were screened. Between the CPB2 + si-*S100A9* and CPB2 + si-NC groups, 221 significant DEGs were upregulated and 95 significant DEGs were downregulated (Supplementary Table S1). Based on the volcano plot, we can see which genes are upregulated and downregulated. (Fig. 2B). The heat map was plotted using the top 100 genes with the smallest *p*-values (Fig. 2C). The top 5 upregulated and downregulated genes with the smallest *p*-values are given in Table 3.

**Table 3:** Most significantly enriched top 5 up- and downregulated genes

gene_id	gene_name	fc	log_2_(fc)	pval	qval	regulation
ENSSSCG00000028810	ENSSSCG00000028810	7.171726749	2.842320521	1.18271E^−75^	1.45059E^−71^	up
ENSSSCG00000016925	PLK2	2.4379802	1.285686409	1.19393E^−32^	4.88116E^−29^	up
ENSSSCG00000004192	CCN2	4.983093204	2.317041558	1.45433E^−31^	4.45933E^−28^	up
ENSSSCG00000014931	ENSSSCG00000014931	6.126639535	2.615095972	9.317E^−28^	1.90455E^−24^	up
ENSSSCG00000040926	ENSSSCG00000040926	22.83465747	4.513153244	2.20666E^−26^	3.86639E^−23^	up
ENSSSCG00000015781	ENPP6	0.289999673	− 1.785876821	4.3553E^−56^	2.67089E^−52^	down
ENSSSCG00000002873	GPI	0.450993256	− 1.148822234	2.28034E^−29^	5.59368E^−26^	down
ENSSSCG00000037524	LY6D	0.495922309	− 1.011813968	5.54808E^−22^	7.5608E^−19^	down
ENSSSCG00000018061	ENSSSCG00000018061	0.437708933	− 1.191956268	4.08562E^−18^	3.34068E^−15^	down
ENSSSCG00000038444	ENY2	0.487132376	− 1.037614224	3.77745E^−17^	2.89565E^−14^	down

### GO and KEGG enrichment analyses

**Fig. 3:**
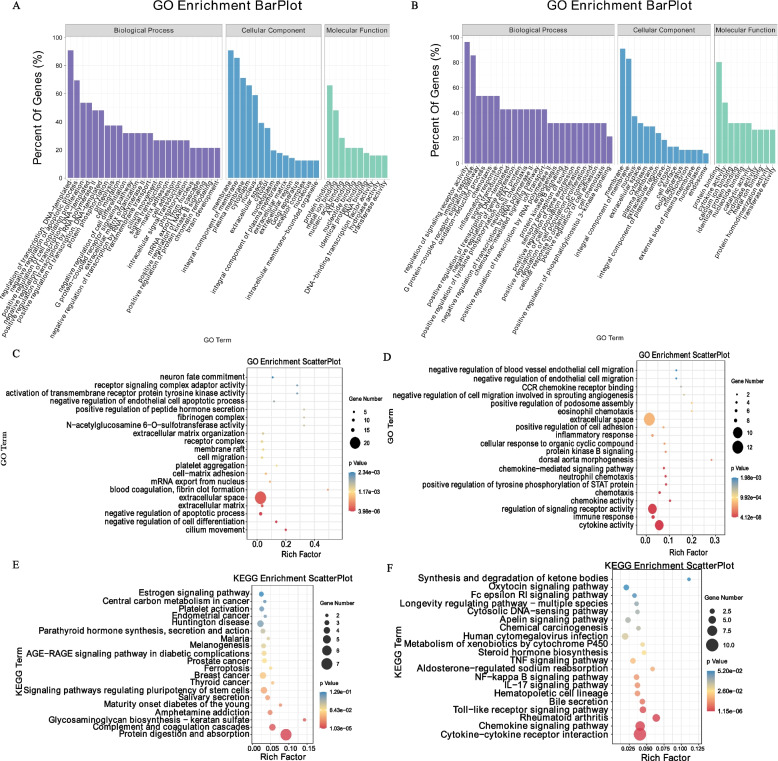
GO enrichment and KEGG pathway analyses of DEGs. **A**, **B** Histogram of up- and downregulated DEGs in GO enrichment analysis. **C**, **D** Scatter plots of Top20 GO enrichment terms for up- and downregulated DEGs. **E**, **F** Scatter plots of Top20 KEGG pathway for up- and downregulated DEGs

A functional enrichment analysis was conducted separately for up- and downregulated DEGs, and the scatter plot was constructed using the top 20 GO terms with the smallest p-values (most significantly enriched) in the enrichment analysis results. The 221 significantly upregulated genes were significantly enriched in 823 BP terms, 158 CC terms, and 249 MF terms (Fig. 3A, Supplementary Table S2). The GO terms that were significantly enriched were those related to cilia movement, negative regulation of cell differentiation, negative regulation of apoptotic processes, extracellular matrix, extracellular space, blood clotting, and fibrin clot formation (Fig. 3C, Supplementary Table S3). Further, 95 significantly downregulated genes were significantly enriched in 541 BP terms, 102 CC terms, and 182 MF terms (Fig. 3B, Supplementary Table S4). The significantly enriched GO terms were cytokine activity, immune response, regulation of signaling receptor activity, chemokine activity, chemotaxis, and signaling pathways associated with inflammatory responses (Fig. 3D, Supplementary Table S5). In addition, KEGG pathway analysis revealed that the significant KEGG signaling pathways in which the upregulated genes were enriched were protein digestion and absorption, complement and coagulation cascades, glycosaminoglycan biosynthesis-keratan sulfate, amphetamine addiction, etc. (Fig. 3E, Supplementary Table S6). The significantly enriched KEGG pathways for downregulated genes included cytokine-cytokine receptor interaction, chemokine signaling pathway, rheumatoid arthritis, toll-like receptor signaling pathway, and bile secretion (Fig. 3F, Supplementary Table S7). Bioinformatic analyses indicated that *S100A9* knockdown regulated CPB2-induced apoptosis and inflammatory response in IPEC-J2 cells.

### PPI network analysis of DEGs

**Fig. 4:**
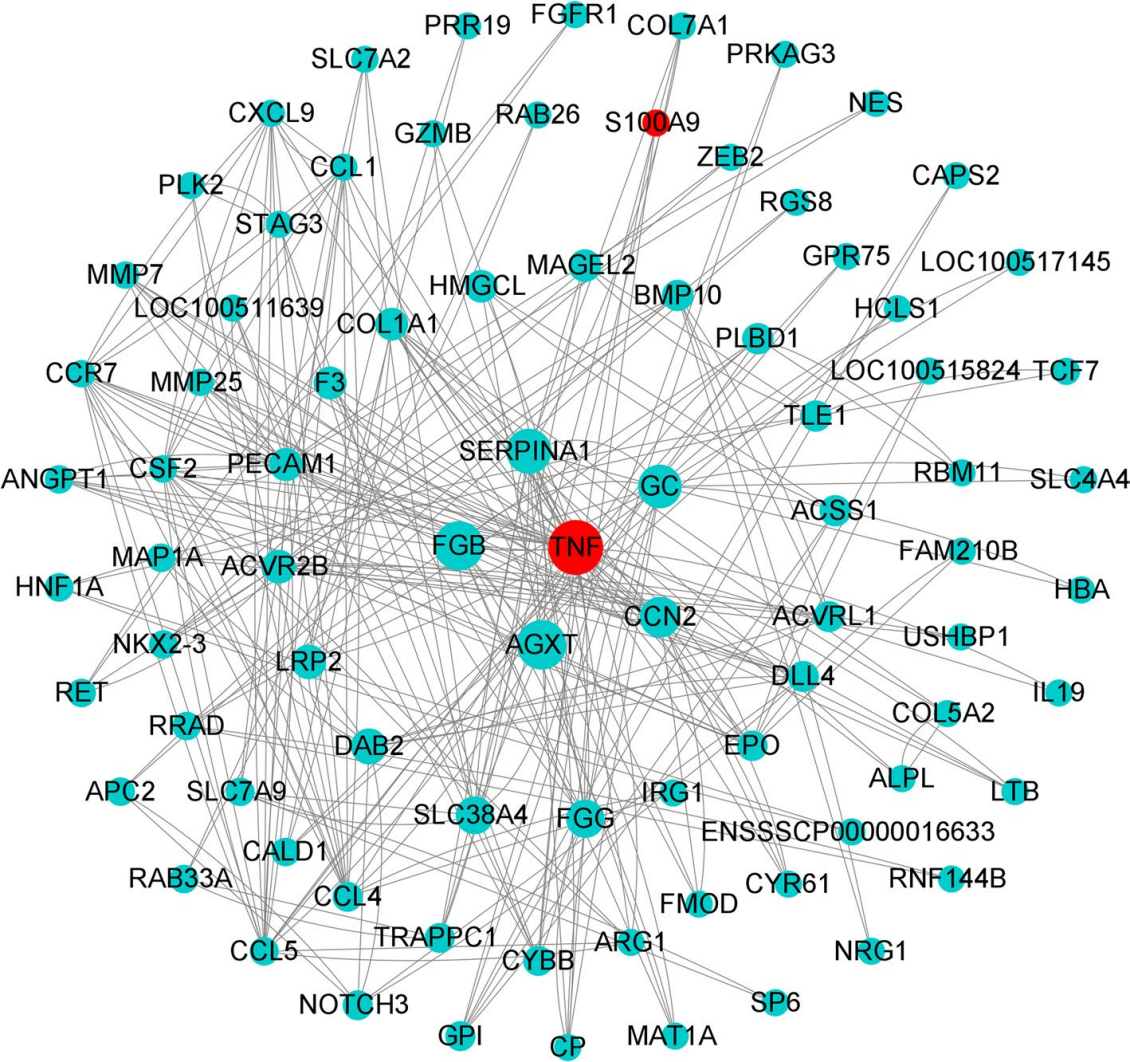
The PPI network analysis of DEGs. Note: The bigger the circle, the higher the BC score with higher degree of association with other nodes. The more the nodes are connected to each other, with stronger node-to-node association

The PPI network of DEGs revealed that 84 DEGs were strongly associated, including 260 edges. Of them, the TNF protein BC value (3165.33) was the highest and the most critical node in the PPI network, which may play a key role in the immune inflammatory response regulated by S100A9 (Fig. 4). The descending order of BC values was for AGXT (2542), FGB (2520), SERPINA1 (2052), GC (1880), and CCN2 (1553.9). The MCODE analysis revealed four protein cluster subnetworks (subnetwork) with high degree of association in the PPI network (Supplementary Fig. 2A–D). Clusters 1 (score 6.857), 2 (score 4), 3 (score 3), and 4 (score 3) contained 8 nodes and 48 edges, 4 nodes and 12 edges, 3 nodes and 6 edges, and 3 nodes and 6 edges, respectively. The MCC algorithm was used to screen the top 5 hub genes from the PPI network, namely, *TNF*, *CCL1*, *CCR7*, *CSF2*, and *CXCL9*, which are involved in immune regulation and inflammatory processes.

### Assessment of differential mRNA levels using RT-qPCR

**Fig. 5:**
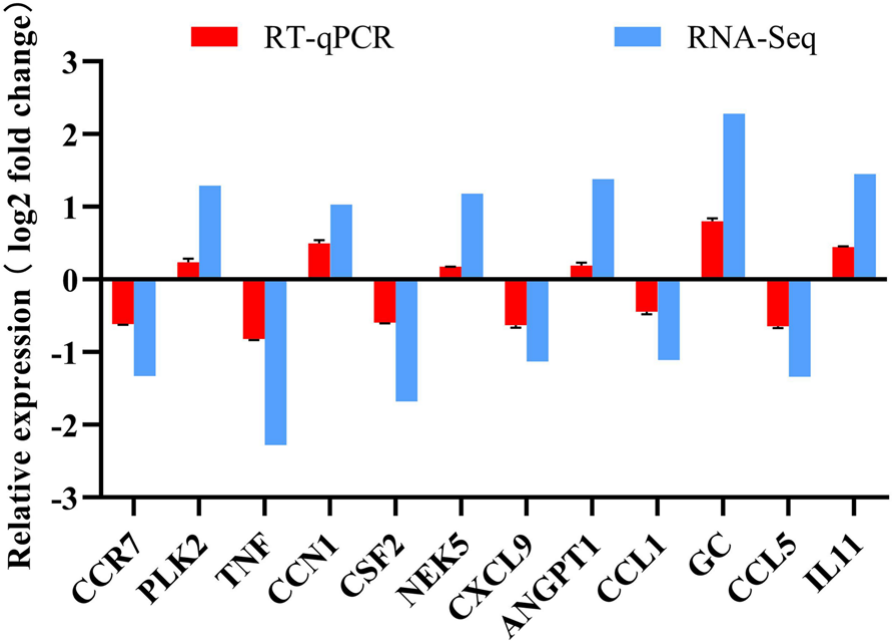
Validation of RNA-Seq using RT-qPCR

The RNA-Seq results were confirmed using RT-qPCR. The expression levels of *CCR7*, *TNF*, *CSF2*, *CXCL9*, *CCL1* and *CCL5* genes were lower in CPB2 + si-*S100A9* samples, while the expression levels of *PLK2*, *CCN1*, *NEK5*, *ANGPT1*, *GC* and *IL11* genes were higher in CPB2 + si-*S100A9* samples (Fig. 5). The RT-qPCR results were consistent with the RNA-Seq analysis results, which proved that the RNA-Seq sequencing results were reliable.

### *S100A9* inhibition alleviated CPB2-induced apoptosis in IPEC-J2 cells

**Fig. 6:**
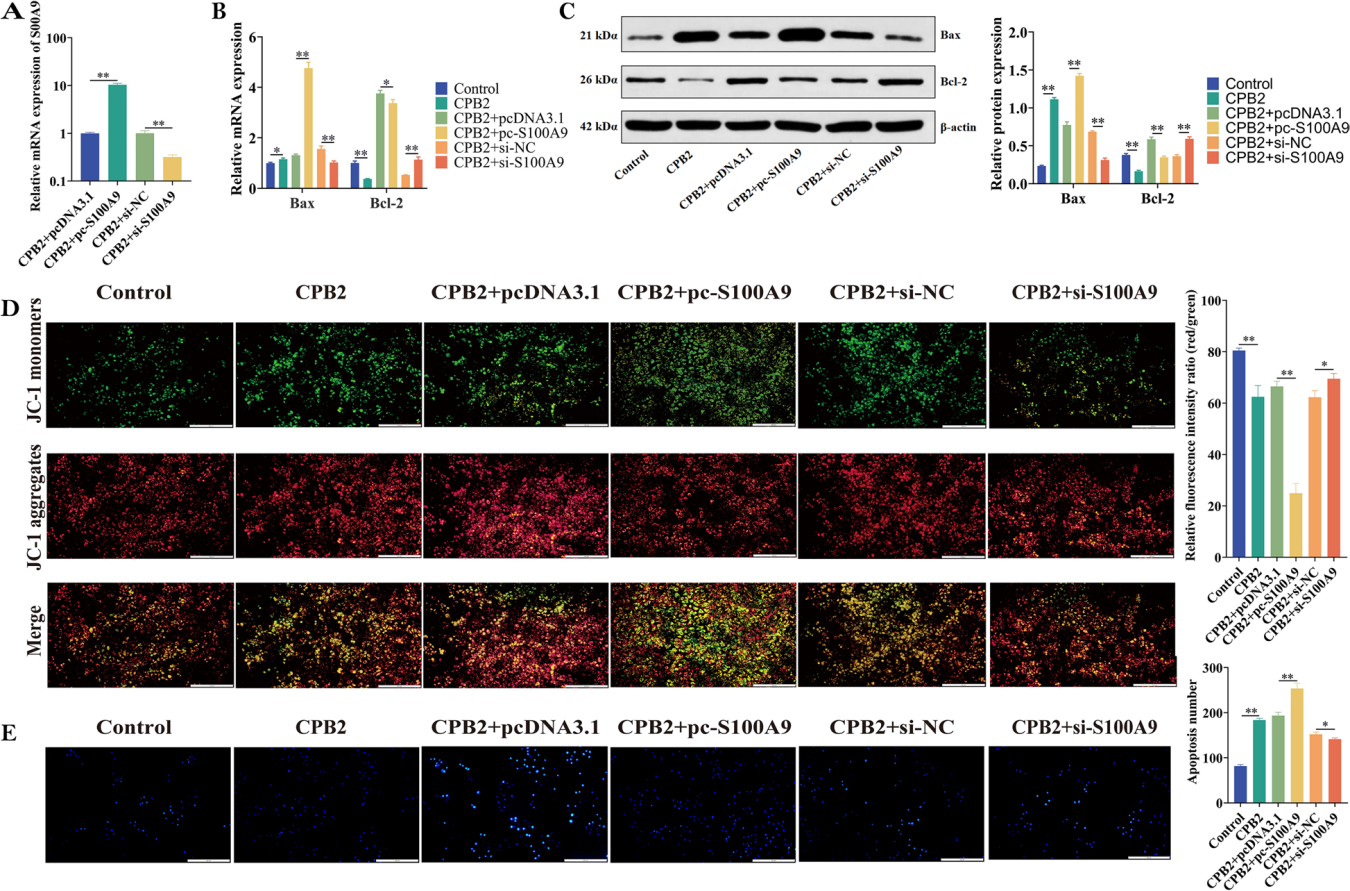
Effects of *S100A9* on CPB2-induced apoptosis in IPEC-J2 cells. **A** *S100A9* transfection efficiency assay. **B** The mRNA levels of *Bax* and *Bcl-2* were detected using RT-qPCR. **C** The protein levels of Bax and Bcl-2 were detected using western blot analysis. **D** Mitochondrial membrane potential assay. The enhanced green fluorescence of the JC-1 fluorescent probe indicated increased apoptosis (low mitochondrial membrane potential), and the enhanced red fluorescence indicated that the mitochondrial membrane potential was high. Scale bar = 200 µm. **E** Hoechst 33,258 staining to detect apoptosis. Scale bar = 200 µm. Blue fluorescence indicates apoptotic cells. *P < 0.05, **P < 0.01

GO enrichment analysis revealed that significantly upregulated genes were enriched in GO terms associated with apoptotic processes. To further investigate the effect of *S100A9* on CPB2-induced apoptosis, we transfected 80% confluent IPEC-J2 cells with *S100A9* overexpression vector pc-*S100A9* and overexpression negative control pcDNA3.1 or *S100A9* interference vector si-*S100A9* and interference negative control si-NC. After transfection, the cells were incubated with 20 μg/mL CPB2 for 24 h. The transfection efficiency of *S100A9* was assessed using RT-qPCR at the end of the treatment (Fig. 6A), and the overexpression and interference of *S100A9* were observed to be successful. Further, the relative mRNA levels of *Bax* and *Bcl-2* were detected using RT-qPCR (Fig. 6B). The *Bax* mRNA levels were significantly higher (*P* < 0.05), and *Bcl-2* mRNA levels were significantly lower in the CPB2 group than those in the control group (*P* < 0.01). Compared with the transfection with pcDNA3.1, *Bax* was significantly upregulated (*P* < 0.01), and *Bcl-2* was significantly downregulated in the pc-*S100A9* group (*P* < 0.05). The mRNA level of *Bax* was significantly lower and that of *Bcl-2* was significantly higher in the si-*S100A9* group than that in the si-NC group (*P* < 0.01). Meanwhile, western blot analysis revealed that the expression patterns of Bax and Bcl-2 proteins were consistent with the results of RT-qPCR (Fig. 6C, Supplementary Fig. 3).

In the early stages of apoptosis, lower mitochondrial membrane potentials are landmark events. The changes in mitochondrial membrane potential were studied in IPEC-J2 cells. The relative fluorescence intensity ratio (red/green) was significantly reduced in the CPB2-induced IPEC-J2 cell injury model compared with the control group (*P* < 0.01); it was significantly reduced after pc-*S100A9* transfection compared with the pcDNA3.1 group (*P* < 0.01). It was higher in the si-*S100A9* group than that in the si-NC group (*P* < 0.05; Fig. 6D).

Apoptosis was detected using Hoechst 33,258 staining. The results revealed that the apoptosis of IPEC-J2 cells was significantly increased due to CPB2 treatment (*P* < 0.01). The number of apoptotic cells increased significantly due to pc-*S100A9* transfection compared with that due to pcDNA3.1 transfection (*P* < 0.01), whereas the number of apoptotic cells due to si-*S100A9* transfection was lower than that in the si-NC group (*P* < 0.05; Fig. 6E). These results indicated that *S100A9* promoted the CPB2-induced apoptosis of IPEC-J2 cells.

### Superoxide dismutase (SOD) activity assay

**Fig. 7:**
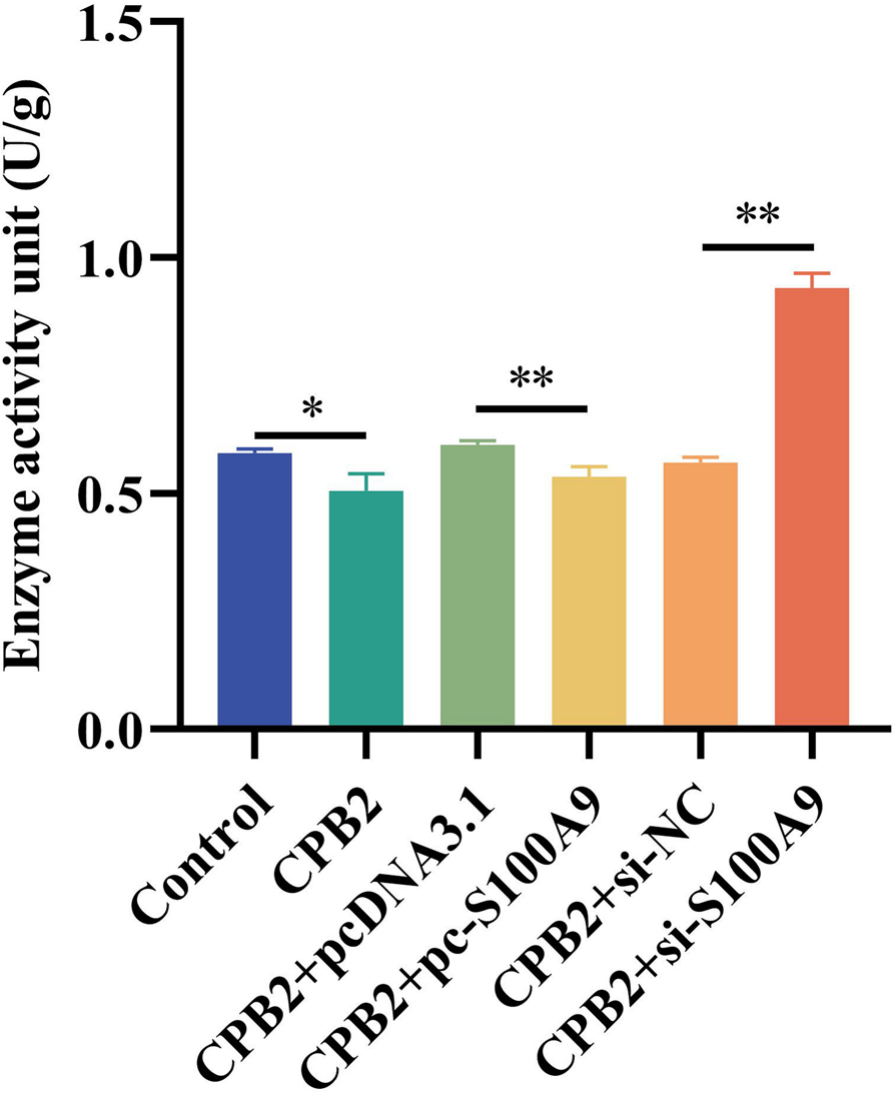
The SOD activity assay of CPB2-treated IPEC-J2 cells. *P < 0.05, **P < 0.01

Living organisms produce SOD, which eliminates harmful substances produced during metabolism. RNA-Seq analysis revealed that CPB2-induced downregulated DEGs in si-*S100A9* cells were significantly enriched in GO terms such as immune response and inflammatory response. We measured SOD enzyme activity to assess cellular damage and to investigate the role of *S100A9* in CPB2-induced inflammatory response in IPEC-J2 cells. The results revealed that under CPB2 induction, SOD activity was significantly lower in the CPB2 group than that in the control group (*P* < 0.05). SOD activity was significantly lower in the pc-*S100A9* group than that in the pcDNA3.1 group (*P* < 0.01). It was extremely significantly higher in the si-*S100A9* group than that in the si-NC group (*P* < 0.01; Fig. 7). Our result indicated that the downregulation of *S100A9* could attenuate cellular damage due to oxidative stress.

## Discussion

*S100A9* is considered a damage-associated molecular pattern [29] and plays an important role in the regulation of inflammatory processes and immune responses [30]. The high level of *S100A9* at the site of inflammation acts as a sensitizing factor to activate neutrophils and causes inflammatory diseases [29]. It is currently known that *S100A9* plays a key role in various inflammatory diseases such as tumors and rheumatoid arthritis. However, to the best of our knowledge, this is the first study to report the molecular mechanism of regulation of piglet diarrhea by *S100A9*. A previous study reported a 95-fold increase in S100A9 protein levels in the bile of patients with primary sclerosis cholangitis, and *S100A9* levels correlate with disease severity [31, 32]. Mosca et al. [33] studied the changes in *S100A9* in patients with inflammatory muscle diseases, and immunostaining revealed significantly higher levels of S100A9 protein in diseased tendons. Moreover, it is reported that *S100A9* promotes endotoxin-induced shock and has been identified as an amplifier of autoimmune and inflammatory responses [34, 35]. We used CPB2-treated IPEC-J2 cells to explore the expression of *S100A9*. Our findings revealed that *S100A9* expression was significantly elevated in IPEC-J2 cells after CPB2 treatment. Therefore, *S100A9* may be a key molecule in regulating the inflammatory response in IPEC-J2 cells after CPB2 treatment.

In our study, we first downregulated the expression of *S100A9* in IPEC-J2 cells, constructed an injury model using CPB2 toxin, and performed transcriptome sequencing. Overall, 316 DEGs were screened using the criteria of |log_2_(fold change)|≥ 1 and *p*-value < 0.05. In total, 221 DEGs were upregulated and 95 DEGs were downregulated in the *S100A9* interference group compared with the negative control group. The significantly enriched GO terms were cilium movement, negative regulation of cell differentiation, negative regulation of apoptotic process, cytokine activity, immune response, etc. KEGG signaling pathway analysis revealed that DEGs were significantly enriched in protein digestion and absorption, cytokine–cytokine receptor interaction, etc. As important regulators of the immune response, cytokines can be involved in gene transcriptional regulation and inflammatory responses through various interaction networks, including kinase and signal transduction [36, 37]. Chemokines are essential members of the defense system, which control immune cell migration and direct cell motility during the inflammatory response and immune process; several chemokines can aggravate the pathological state of the inflammatory response [38, 39]. Viemann et al. [40]reported that *S100A9* promotes the expression of proinflammatory chemokines to induce inflammatory responses. In the human immune system, *CCR7*, as an important chemokine, regulates the transport process of immune cells during immune and inflammatory responses [41]. According to some studies, *CCR7* may contribute to the CPB2-induced inflammatory injury in IPEC-J2 cells, and its expression is downregulated in *S100A9* knockdown cells; therefore, *S100A9* may be a prognostic marker. Furthermore, it is worth noting that the sequencing results revealed that the downregulated genes were significantly enriched in GO terms of immune response. This suggested that *S100A9* regulates CPB2-induced inflammatory damage in IPEC-J2 cells, potentially by regulating downstream target genes.

PPI networks are essential for studying biologically active processes in cells [42], where prediction of PPI networks is useful for understanding cellular processes in organisms [43]. In this study, by the construction of a PPI network of DEGs, a protein cluster with a score of 6.857 was observed to be the most critical functional module in the PPI network, and TNF was the most critical node. TNF, as an intracellular regulatory factor, is a signaling protein in autoimmune diseases [44]. *TNF* is reported to mediate a broad range of inflammatory diseases and is a regulator of inflammation and inflammation-related diseases [45, 46], such as inflammatory bowel disease, cardiovascular disease, and rheumatoid arthritis. In addition, it promotes the release of inflammatory cytokines *IL6* and *IL8*, which act together with several cytokines [47]. In our study, the DEGs *IL11* and *IL19* were upregulated. Further, our sequencing results revealed that *CCL1*, *CCR7*, *CSF2*, and *CXCL9* genes were downregulated in *S100A9* knockdown cells and were mostly enriched in the cytokine–cytokine receptor interaction signaling pathway. Notably, these genes are present in the major protein cluster subnetwork, which indicated that these genes play an important role in the *S100A9*-mediated inflammatory response in IPEC-J2 cells.

Functional enrichment analysis revealed that DEGs were associated with the negative regulation of the apoptotic pathway. Therefore, we studied the effect of *S100A9* on CPB2-induced apoptosis in IPEC-J2 cells by multiple assays and observed that *S100A9* overexpression promoted apoptosis under CPB2 treatment, whereas *S100A9* downregulation inhibited apoptosis. *S100A9* is reported to have apoptosis-inducing activity on several cell types, which may be achieved by regulating the balance between pro- and antiapoptotic proteins [48]. *S100A9* is currently the most widely studied marker in human diseases; however, its role in porcine apoptosis is not extensively studied. A study reported that *S100A9* upregulation exhibited apoptosis-inducing activity, and *S100A9* downregulation attenuated apoptosis in esophageal squamous carcinoma cells [49]. Similarly, Ghavami et al. [50] reported that S100A8/A9 decreased MCF7 and SHEP cell viability in a concentration- and time-dependent manner and exhibited apoptosis-inducing activity. Consistent with our results, Nakatani et al. [51] reported that *S100A8/A9* could induce apoptosis by binding to MM46 cells under the regulation by zinc ions.

SOD is the main active molecule for scavenging reactive oxygen species in animals [52]. Increasing SOD levels can alleviate oxidative stress damage caused by reactive oxygen species; therefore, SOD can play a protective role for the organism [53, 54]. Schilrreff et al. [55]reported anti-inflammatory and antioxidant activities of orally administered nanosomes assembled with SOD. Similarly, Satomi et al. [56] reported that SOD activity was higher in cancerous cells than in normal tissues, and SOD activity was positively correlated with cancer progression. These studies suggested that SOD can be used as an analytical index for further analysis of cellular damage. Accordingly, we assessed the effect of *S100A9* on CPB2-induced cytotoxicity and cell damage in IPEC-J2 cells by measuring the enzymatic activity of SOD in each experimental group. Our study revealed that SOD activity was elevated after *S100A9* downregulation under CPB2 treatment, which could alleviate oxidative stress damage.

## Conclusion

Using RNA-Seq, we screened the downstream key genes of *S100A9* regulating inflammatory response and revealed the key role of *S100A9* in CPB2-induced inflammatory injury in IPEC-J2 cells. *S100A9* participated in CPB2-induced inflammatory response in IPEC-J2 cells by regulating the expression of downstream target genes, namely, *TNF*, *CCL1*, *CCR7*, *CSF2*, and *CXCL9*. *S100A9* promoted apoptosis and exacerbated oxidative stress injury. This paper laid the foundation for further studying the molecular regulatory mechanism underlying piglet diarrhea.

## Supplementary Information

**Additional file 1:** **Supplementary Figure 1.** (A) Machine exposure images, (B) Manually exposed image, (C) S100A9 film, (D) β-actin film.

**Additional file 2:** **Supplementary Figure2.** The PPI network analysis of DEGs. (A–D) Subnetwork of protein clusters with higher degree of association in the complete PPI network.

**Additional file 3:** **Supplementary Figure 3.** (A) Machine exposure images, (B) Manually exposed image, (C) Bax film, (D) Bcl-2 film, (E) β-actin film.

**Additional file 4:** **Supplementary Table S1.**

**Additional file 5:** **Supplementary Table S2.**

**Additional file 6:** **Supplementary Table S3.**

**Additional file 7**: **Supplementary Table S4.**

**Additional file 8:** **Supplementary Table S5.**

**Additional file 9:** **Supplementary Table S6.**

**Additional file 10:** **Supplementary Table S7.**

## Data Availability

The data used in this study are presented in this study and supplementary materials, and the RNA-Seq data have been uploaded to the SRA database with the Bioproject accession number PRJNA856459. The link for data is as follows: https://dataview.ncbi.nlm.nih.gov/object/PRJNA856459?reviewer=1ag61ljj6npjjmb1e1sr487a2l
