# Supplementary material for: S100A9 plays a key role in Clostridium perfringens beta2 toxin-induced inflammatory damage in porcine IPEC-J2 intestinal epithelial cells
Source: BMC Genomics. 2023 Jan 12;24:16. doi: 10.1186/s12864-023-09118-6 (PMC9835341; doi:10.1186/s12864-023-09118-6)
Supplement: Supplementary file 2 — Additional file 2: Supplementary Figure2. The PPI network analysis of DEGs. (A–D) Subnetwork of protein clusters with higher degree of association in the complete PPI network. [file 12864_2023_9118_MOESM2_ESM.pdf]

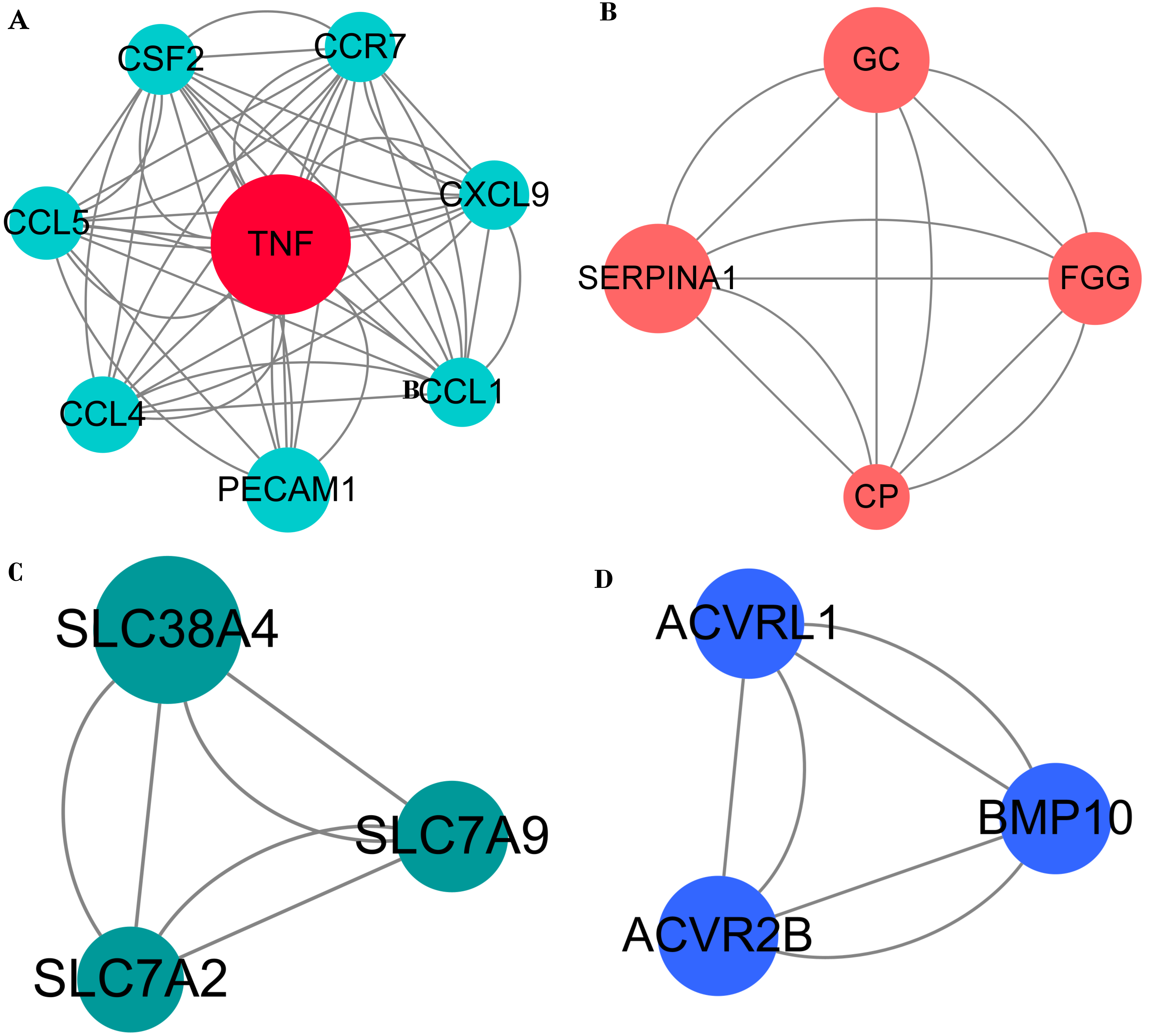

Supplementary Figure2 The PPI network analysis of DEGs. (A–D) Subnetwork of protein clusters with higher degree of association in the complete PPI network.
Note: The bigger the circle, the higher the BC score with higher degree of association with other nodes. The more the nodes are connected to each other, with stronger node-to-node association.
