# Supplementary material for: S100A9 plays a key role in Clostridium perfringens beta2 toxin-induced inflammatory damage in porcine IPEC-J2 intestinal epithelial cells
Source: BMC Genomics. 2023 Jan 12;24:16. doi: 10.1186/s12864-023-09118-6 (PMC9835341; doi:10.1186/s12864-023-09118-6)
Supplement: Supplementary file 3 — Additional file 3: Supplementary Figure 3. (A) Machine exposure images, (B) Manually exposed image, (C) Bax film, (D) Bcl-2 film, (E) β-actin film. [file 12864_2023_9118_MOESM3_ESM.pdf]

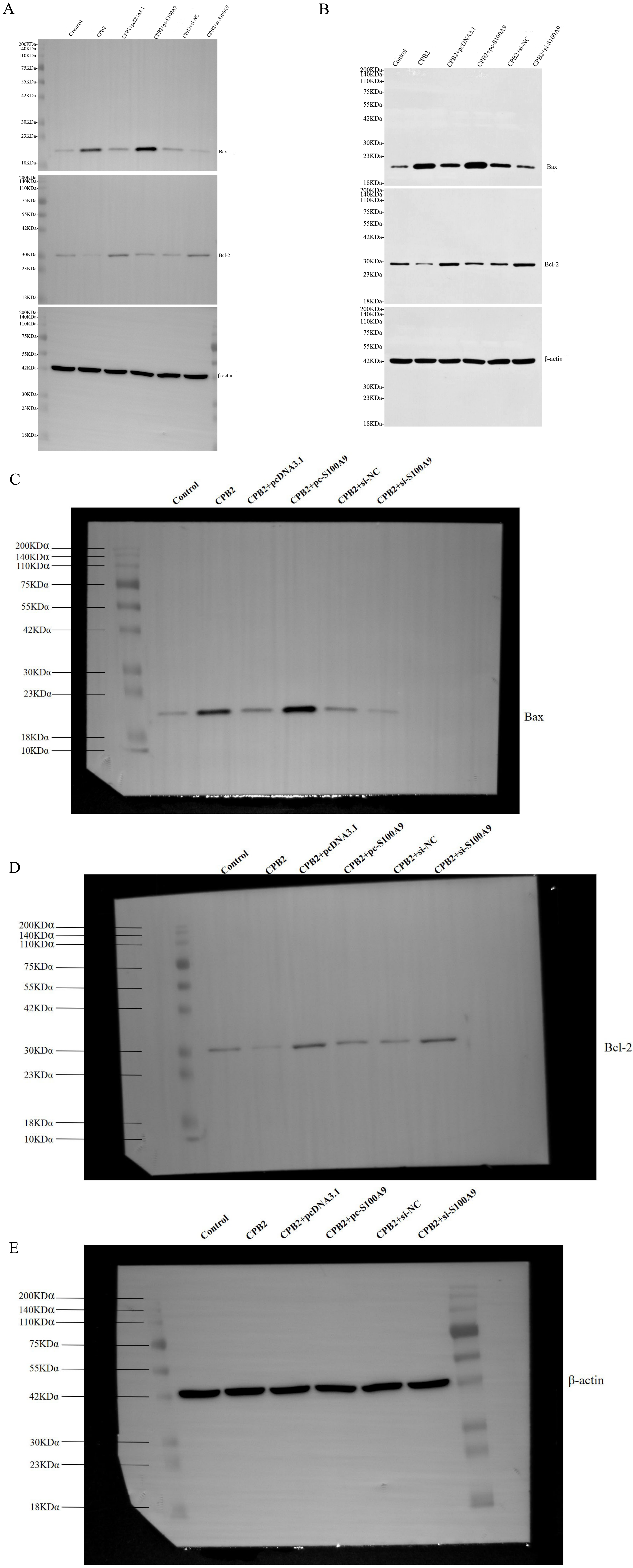

Supplementary Figure 3 (A) Machine exposure images, (B) Manually exposed image, (C) Bax film, (D) Bcl-2 film, (E) β-actin film

Note: Figure B shows the manual exposure image, because the marker itself does not emit light, and the molecular weight size can only be marked manually on top of the film. Second, during manual exposure, we turn up the brightness of all the strips as a whole in order to make them clearer, which has no effect on the analysis results. The images used in the article were taken from Figure B.
